# Exposure Assessment and Risk Characterization of Aflatoxin M1 Intake through Consumption of Milk and Yoghurt by Student Population in Serbia and Greece

**DOI:** 10.3390/toxins11040205

**Published:** 2019-04-05

**Authors:** Bozidar Udovicki, Ilija Djekic, Eleni P. Kalogianni, Andreja Rajkovic

**Affiliations:** 1 Department of Food Safety and Quality Management, Faculty of Agriculture, University of Belgrade, 11080 Zemun-Belgrade, Serbia; bozidar.udovicki@agrif.bg.ac.rs (B.U.); idjekic@agrif.bg.ac.rs (I.D.); 2 Department of Food Technology, Alexander Technological Educational Institute of Thessaloniki, 57400 Thessaloniki, Greece; elekalo@food.teithe.gr; 3 Department of Food Technology, Food Safety and Health, Faculty of Bioscience Engineering, Ghent University, B-9000 Ghent, Belgium

**Keywords:** milk, aflatoxin M1, exposure assessment, risk characterization, Monte Carlo, HCC, MOE

## Abstract

The objective of this research was to perform an exposure assessment of aflatoxin M1 (AFM1) intake through the consumption of milk and yoghurt by the student population in Serbia and Greece. A food consumption survey of milk and yoghurt was performed during the first half of 2018 in the two countries with at least 500 interviewees (aged between 18 and 27 years) per country, covering their dietary habits and body weight based on one-day and seven-day recall methods. Values for the concentration of AFM1 were extracted from published research. Finally, a Monte Carlo analysis of 100,000 iterations was performed to estimate the intake of AFM1 from the consumption of the two dairy products. Results revealed that the estimated average exposure of students to AFM1 was in the range of 1.238–2.674 ng kg^−1^ bw day^−1^ for Serbia, and 0.350–0.499 ng kg^−1^ bw day^−1^ for Greece, depending on the dietary recall method employed. High estimations for hepatocellular carcinoma (HCC) cases/year/10^5^ individuals, depending on the prevalence of Hepatitis B virus surface antigen positive individuals (HBsAg+), were 0.0036–0.0047 and 0.0007–0.0009 for Serbia and Greece, respectively. Presented Margin of Exposure (MOE) and Hazard Index (HI) values indicate increased risk from exposure to AFM1, particularly in Serbia.

## 1. Introduction

Aflatoxins (AFs), secondary metabolites of some *Aspergillus* spp. members, are a group of potent carcinogenic and teratogenic compounds that occur in various agricultural commodities. Aflatoxin M1 (AFM1) is a hydroxylated form of aflatoxin B1 (AFB1), the most prevalent and most potent member of this group, which is, when ingested and transformed by the liver to AFM1, easily secreted through milk [1,2]. Aflatoxin M1 differs structurally from the parent molecule in that a hydroxyl group is present in the difuran moiety of its molecule. Like AFB1, it is known that the target organ of AFM1 toxicity is the liver. The International Agency for Research on Cancer (IARC) stated that there was enough evidence for the carcinogenicity of AFM1 in experimental animals. It has also reported that, even though there was inadequate evidence for the carcinogenicity in humans, AFM1 was assigned to the IARC’s carcinogenicity group 2B due to its similarity with AFB1 (Group A member) in structure, activity, and other relevant evidence [3]. The carcinogenic potency of AFM1 is approximately 2% to 10% of AFB1 [4]. Regarding this carcinogenic effect, the essential role of the immune system related to the incidence, severity, and outcome of infectious diseases indicate that, through its reported effect on the immune system and micronutrients, AFs may also affect the epidemiology of numerous diseases and health risks [5].

Concerning the exposure to AFM1, high milk consumption appears to be one of the most important factors by all age groups. [6]. Milk is considered one of the world’s most complete foods. Next to the role of meeting daily nutrient recommendations, intake of milk and dairy products was associated with a neutral or reduced risk of type 2 diabetes and a reduced risk of cardiovascular disease, particularly stroke [7]. Furthermore, the evidence suggested a beneficial effect of milk and dairy intake on bone mineral density but no association with the risk of bone fracture [7]. Bearing in mind the health risks associated with AFM1, numerous countries have established permissible limits for maximum level (ML) of AFM1 in milk. The European Commission had set an ML value of 0.05 µg kg^−1^ for raw milk, heat-treated milk and milk for the production of milk-based products [8], whereas in Serbia this value is 0.25 µg kg^−1^ [9].

Students (aged between 18 and 27 years) were chosen for this exposure assessment as they are the source of development in every country. Higher education provides considerable value to individuals, to the economies where educated individuals live and work, and society in general [10]. As intake values of AFM1 were found to decrease with age [11,12], AFM1 intake by student population is of particular interest. Namely, the population of students is a population at the critical stage of transitioning from dietary habits, mostly controlled by parents and preschools, into independent nutritional practices [13], which can last a lifetime.

Therefore, the objective of the present study is to evaluate exposure to AFM1 of university students in Serbia and Greece, via milk and yoghurt consumption, and at the same time to evaluate the potential risk, applying different risk-assessment scenarios.

## 2. Results and Discussion

### 2.1. Demographic Profile and Consumption Patterns

Overall, the demographic profile (Table 1) shows that the female population prevailed (58.0%). Male/female sample population ratio is consistent with the official national data in Greece [14], whereas in Serbia the female population size was higher in the sample population [15]. Distribution of respondents regarding their age showed that slightly more than half of the sample was younger than 22 years. Average body weight was around 68 kg.

Table 2 displays the frequency of consumption of milk in Serbia and Greece where most of the young respondents confirmed that they drink milk on at least a weekly basis. When they consume milk, the average quantity is between 200 mL and 300 mL in Serbia (52.1% of sample population) and in Greece (29.5% of sample population). When they consume yoghurt, the average quantity is between 200 mL and 300 mL in Serbia (45.2% of sample population) and in Greece (36.8% of sample population). In addition, this study showed a significant association between countries and consumption patterns (*p* < 0.05), for both milk and yoghurt. No statistically significant differences were observed for gender and age (*p* > 0.05).

Depending on the number of intake days considered, the average daily consumption of both milk and yoghurt was in the range of 310–550 mL and 480–700 mL for Serbia and Greece, respectively. The 95th percentile of milk and yoghurt intake (high consumers) was in the range of 770–1080 mL and 930–2600 mL for Serbia and Greece, respectively.

### 2.2. Exposure Assessment and Risk Characterization

Prevalence of AFM1 in Serbia (Table 3) was 85.7% (*N* = 1793) with the mean value of 0.049 μg kg^−1^ and in the range of 0.003 (lowest reported LOD)–0.319 μg kg^−1^ [11,16,17,18,19]. As there was only one published paper on AFM1 in milk from Greece in this decade, bibliographic research included earlier studies and conference papers. Prevalence of AFM1 in Greece (Table 3) was 55.8% (*N* = 285) with a mean value of 0.011 μg kg^−1^ (calculated as Pooled Mean of reported means), and in the range of 0.005 (LOD)–0.05 μg kg^−1^ [20,21,22]. Considering that most reported values are well below the permitted ML and that the European Union (Greece is an EU member) has the best global food safety systems, it would be safe to presume that there was no significant increase in AFM1 contamination of milk in Greece and that reported values can be used in this exposure assessment.

**Figure 1:**
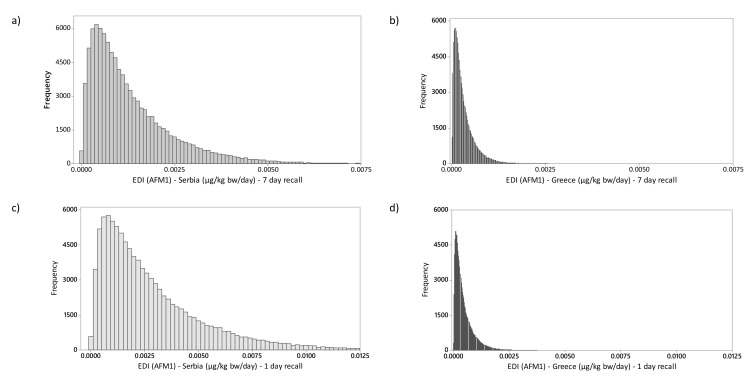
Comparison of estimated total daily intake of mycotoxins after a Monte Carlo analysis of 100,000 simulations. (**a**) AFM1 Serbia (7-day recall); (**b**) AFM1 Greece (7-day recall); (**c**) AFM1 Serbia (1-day recall); (**d**) AFM1 Greece (1-day recall).

Estimation of a mean AFM1 exposure (Figure 1, Table 4) was in the range of 1.238–2.674 ng kg^−1^ bw day^−1^ for Serbia, and 0.350–0.499 ng kg^−1^ bw day^−1^ for Greece, depending on the number of intake days considered.

For quantitative cancer risk assessment percentages of HBsAg+ individuals of 1.2% (low estimate) and 2.6% (high estimate) reported for the European Region were considered [23]. High estimations for hepatocellular carcinoma cases/year/10^5^ individuals (based on 1-day recall dietary survey), depending on the HBsAg+ prevalence (Table 4), were 0.0036–0.0047 and 0.0007–0.0009 for Serbia and Greece, respectively. Presented Margin of Exposure (MOE) and Hazard Index (HI) values (Table 4) indicate increased risk from exposure to AFM1, especially in Serbia.

Early estimation of AFM1 intake performed in 2001 by the Joint FAO/WHO Expert Committee on Food Additives (JECFA) was calculated to be 6.8 ng per person per day (approximately 0.11 ng kg^−1^ bw day^−1^) for the European type diet [24]. This estimation was calculated based on the European regional consumption of both milk and milk products of 340 g per person per day (the Global Environment Monitoring System (GEMS)/Food regional diets data [25]) and the weighted mean concentration of AFM1 in the milk of 0.023 μg kg^−1^. Considering the deterministic approach of this estimation and considerably higher reported consumption of milk obtained by this study, we can say, with some level of uncertainty, that exposure to AFM1 in Greece falls into a similar range. On the other hand, exposure to AFM1 in Serbia was considerably higher.

Cano-Sancho et al. [26] estimated the exposure of the adult Catalonian population (20 to 65 years old) to be 0.039 ng kg^−1^ bw day^−1^. The mean milk intake was 305 mL day^−1^ (750 mL day^−1^ for 95th percentile). The First French Total Diet Study presented an estimated average intake of AFM1 in the adult French population of 0.09 ng kg^−1^ bw day^−1^, with the highest value of 0.21 ng kg^−1^ bw day^−1^ [27]. Shundo et al. [28] reported intake of AFM1 by the Brazilian adult population of 0.08 ng kg^−1^ bw day^−1^ based on milk consumption of 350 mL. These estimations were lower than the ones presented in this study. However, they are mostly based on a deterministic approach; milk consumption reported was significantly lower from milk consumption obtained by this study, and calculations were made for a general adult population.

Before 2012, studies on the occurrence of AFs in Serbia have shown no or a low presence of AFs in various food and feed commodities. However, in 2012 Serbia had severe and prolonged drought throughout spring and summer months which most likely has contributed to the high contamination frequency and concentration of AFs in maize and subsequently in milk and dairy products [29]. Similar weather conditions were recorded again in 2015, leading to an increased occurrence of AFM1 [30]. However, as reported by a later study [31], the Serbian dairy industry has responded responsibly and implemented preventive measures to ensure higher milk safety. Kos et al. [18] estimated exposure to AFM1 in Serbia through milk intake in 2013 using obtained mean concentration of AFM1 of 0.21 μg kg^−1^ and a deterministic approach. Results showed milk intake between 100 and 440 mL for the female and male population aged 15–25 years, and exposure to AFM1 in the range of 0.41–1.26 ng kg^−1^ bw day^−1^ for the female and male population. Maximal intake reported in this study was 2.39 ng kg^−1^ bw day^−1^ for the female population and 7.18 ng kg^−1^ bw day^−1^ for the male population. Based on the milk intake data reported in a previous study, Milicevic et al. [16] calculated exposure to AFM1 through heat-treated milk in 2015–2016 in the range of 0.07–0.21 ng kg^−1^ bw day^−1^ for the female and male population aged 15–25 years. Reported HI values were 0.33 for the female population and 0.98 for the male population. Taking into consideration represented exposure assessment, results from Serbia are more comparable to the ones obtained from regions with climate conditions suitable for AF production. In a study from 2018, estimated AFM1 intake from milk was in the range of 0.8 (average intake) and 1.2 (high intake) in the adult population of Kenya [32]. This assessment was based on the average milk intake of approximately 440 mL and mean contamination level of 0.105 μg kg^−1^. The estimated number of hepatocellular carcinoma cases/year/10^5^ individuals was 0.004.

## 3. Conclusions

Infants and young children are commonly recognized as populations vulnerable to the effects of AFM1, as they consume more milk relative to their body weight than adults. This study identified the student populations in Serbia and Greece as groups particularly vulnerable to AFM1 exposure in recent years. In Serbia, this high exposure is mainly due to recent outbreaks of AFs contamination, combined with higher milk and yoghurt consumption, whereas in Greece the source of AFM1 intake is high milk and yoghurt consumption.

As calculations were performed considering exclusively intake of AFM1 from milk and yoghurt, future studies are needed to assess the contribution of other dairy products and products containing milk as a constituent. As authors acknowledge a bibliographic approach to AFM1 contamination as a study uncertainty, up-to-date assessments of AFM1 contamination in dairy products are also recommended to accurately present exposure to AFM1. In the case of Serbia, this use of bibliographic data for a wider time span has led to a certain level of overestimating exposure in relation to the most recent years, especially if we consider preventive measures implemented by the dairy industry. Research should also incorporate other age categories. Additional dietary intake studies, with a higher number of participants, are needed to confirm high milk intake values obtained in this study.

## 4. Materials and Methods

### 4.1. Consumer Survey

The survey on consumption of dairy products was performed in the first half of 2018 in Serbia and Greece. A questionnaire for direct interviews has been created in line with European Food Safety Authority (EFSA) guidelines on data collection for national food consumption [33].

The tested population sample was predetermined in terms of age (all respondents were students over 18 and under 27 years old) and a number of respondents per country (at least 500). Demographic characteristics were not stratified due to restrictions in resources when interviewing two countries and two universities at the same time. Data collection was performed through personal interviews. As all subjects were 18 years and above interviews were conducted with Verbal Consent of the participants. Participants were informed (orally and via written instruction) about general purpose of the survey and basic principles of anonymity, confidentiality and data protection, Questionnaires were collected by authors with the help of their Master’s students in their home faculties and in major university campuses randomly choosing students, as well as using an existing professional and family network.

A three-section structured questionnaire was developed based on similar risk-assessment/exposure-assessment research and general guidelines proposed by the EFSA [33,34]. The first section (Table 1) covered general demographic information about the respondents, namely, gender, age, and weight (self-reported). The second section examined consumers’ consumption patterns for milk and yoghurt (all types of milk and yoghurt) and covered frequency and quantity consumed. These products are the most consumed dairy products and essential parts of diet in both countries [35,36]. The third section gave the respondents an opportunity to analyze consumption of milk and yoghurt the day before and in the last seven days, giving them the option to state the type of product (e.g., low fat, standard fat, with fruit, etc.) and amount of the consumed products (in mL). The 24-h dietary recall is the most common recall method used [33]. Besides the 24-h dietary recall, the authors decided to have a ‘7-day dietary recall’ in order to cross-check the results and to capture habitual intake of milk. Under certain circumstances, EFSA suggests that it may be more efficient to include more recording or recall days per person to estimate habitual exposure to compounds from foods [33]. Other dairy products (such as cheese, cream, ice-cream, etc.) were not included in this study due to the large technological diversity of these products in the two countries, as AFM1 has the capability to bind with various macromolecules, especially proteins [37]. This can lead to different distribution of AFM1 in products of the same type, depending on technology used.

### 4.2. Exposure Assessment

The Food and Agriculture Organization/World Health Organization (FAO/WHO) [38] defines exposure assessment as a qualitative and/or quantitative evaluation of the likely intake of a chemical agent via food, as well as exposure from other sources, if relevant. This methodology is developed to analyze scientific information, to evaluate the severity and probability of an adverse effect to human health through the consumption of food and thus to provide an association between possible hazards in the food chain and the associated risks to human health.

Two main parameters of the exposure assessment are the amount of food consumed through a specific period and food-contamination data. Consumption data were mathematically treated to represent the average amount (mL) of milk and yoghurt consumed per day based on daily and weekly consumption reported during the field research. A bibliographic review was carried out to obtain preliminary estimates of the average concentration of AFM1 in milk in Serbia and Greece. This review was performed by analyzing published articles. The major sources of information were the scholarly databases Web of Science, EBSCO and ScienceDirect, which identified relevant academic articles published in the domains of AFM1 (more specifically: AFM1 in milk and/or dairy products) as well as countries (more specifically: Serbia and Greece). Average concentrations used in calculations of AFM1 exposure were calculated as Pooled Means of reported means as follows: (N1 × M1 + N2 × M2 + Nn × Mn)/(N1 + N2 + Nn), where N is reported number of samples and M is reported mean of AFM1 concentration in milk. When possible, data from reported studies were additionally parsed (in terms of separating means and sample numbers for various milk types e.g., pasteurized, UHT, etc.). The initial search was limited to the studies published in this decade. However, due to a limited number of published papers, the bibliographic search for Greece was expanded.

Several studies have reported that the fermentation process during yoghurt production and subsequent storage have little or no effect on AFM1 levels in yoghurt [39,40,41]. Therefore, values obtained for AFM1 in milk were used for yoghurt as well.

The exposure to AFM1 through dairy-products consumption was calculated as Estimated Daily Intake (EDI) using data on milk and yoghurt consumption [ng kg^−1^ bw day^−1^], AFM1 concentration and body weight (bw), using the following two formulas, one for ‘one-day recall’, Equation (1), and one for ‘seven-day recall’, Equation (2):

(1) $$\mathrm{EDI}=\frac{\sum_{i=1}^{n}D_{i}}{bw}\times C_{t},$$

(2) $$EDI=\frac{\sum_{i=1}^{n}D_{i}}{7}\times \frac{1}{bw}\times C_{t},$$

where *D_i_* is the reported amount of each dairy products consumed on a daily Equation (1) and weekly basis Equation (2) [mL]. Average daily intake of a seven-day recall was calculated by dividing *D_i_* by seven. Body weight (bw) is expressed in [kg], and *C_t_* is the concentration of AFM1 [ng g^−1^].

### 4.3. Risk Characterization

The bottom-up approach, as defined by the WHO was used in risk characterization. This approach uses exposure levels of diets and contamination levels in foods to predict death and mortality [42]. The JECFA [43] derived an estimation of AFB1 carcinogenic potency based on the synergistic hepato-carcinogenic effects of AFB1 and hepatitis B virus infection. As AFM1 is a metabolite AFB1, it is presumed that AFM1 induces liver cancer by a similar mechanism. The JECFA used the comparative data for carcinogenic potency and assumed that the potency of AFM1 is one-tenth that of AFB1. Thus, the carcinogenic potency of AFM1 was estimated to be 0.001 cancer cases/year/10^5^ individuals per 1 ng kg^−1^ bw day^−1^ in Hepatitis B virus surface antigen negative (HBsAg) individuals and 0.03 cancer cases/year/10^5^ individuals per 1 ng kg^−1^ bw day^−1^ in HBsAg+ individuals [24]. Taking into consideration the prevalence of HBsAg+ individuals in the total population, Carcinogenic Potency (Pcancer) of AFM1 was calculated as follows:

(3) $$Pcancer=0.001\;\times \;\%HBsAg^{-}\;+\;0.03\;\times \;\%HBsAg^{+}$$

Risk of hepatocellular carcinoma (HCC) incidence per year, resulting from dietary AFM1 intake through milk consumption, was calculated from the estimated dietary exposure to AFM1 multiplied by the AFM1 cancer potency as follows:

(4) HCC risk=EDI × Pcancer

Additional risk characterization was performed by calculating MOE and HI for AFM1. For carcinogens, such as AFM1, a Tolerable Daily Intake is generally not determined. It is recommended that the concentration of such compounds in food should be as low as reasonably achievable (ALARA).

The MOE approach, proposed by EFSA to analyze the risk of a compound that is both genotoxic and carcinogenic, uses an animal study reference point to evaluate risk [44]. This reference point is then compared with various dietary intake estimates in humans. To obtain MOE, it is recommended to use the BMD (benchmark dose), the dose that causes a low but measurable response or BMDL10 (benchmark dose lower confidence limit 10%), which is an estimate of the lowest dose that is 95% certain to cause no more than 10% cancer incidence [44]. The MOE represents the ratio between the reference dose and the EDI, and considering overall uncertainties in the interpretation, MOE value equal to or higher than 10,000 would be of little concern from a public health point of view. For the purpose of this paper we used results of the 2-year study in male Fischer rats [45], with the main findings that, in rats fed with a diet containing AFM1 at 50 μg kg^−1^, HCC was detected in 2/18 rats when killed at 21 months. From these results, it was estimated that the potency of AFM1 is 2–10% that of AFB1 [4]. The potency of AFM1 in Fischer rats was calculated as follows: 2/18 risk/(1 mg/lifetime × 21 months/lifetime × 31 days/month)/0.3 kg bw = 0.00057 mg kg^−1^ bw day^−1^ [24]. This value was used as a reference dose for MOE calculation.

Further estimates of safe intake can be determined by dividing the TD50 (the daily dose rate in mg kg^−1^ bw day^−1^ to induce tumours in half of the test animals that would have remained tumour-free at zero doses) by an uncertainty factor of 5000, a value equivalent to a risk level of 1:100,000 [46]. The EDI is then divided by this proposed value (0.2 ng kg^−1^ bw day^−1^) to obtain HI. Generally, HI higher than 1 indicates a risk to consumers.

### 4.4. Statistical Analysis

The Chi-Square test for association was used to discover if there are relationships between milk and yoghurt consumption patterns and demographic characteristics of the sample (gender, age and country). The Mann–Whitney U test has been performed to compare the consumption patterns between two groups-categorical variables, such as country, gender and age. Basic descriptive statistical processing and the Chi-Square test for association were performed using MS Excel (MS Office 10, Redmond, WA, USA). Normality testing and Mann–Whitney U Test for milk and yoghurt intake between genders were performed with the SPSS Statistic software package (SPSS 17.0, SPSS Inc., Chicago, IL, USA).

This study also used the Monte Carlo analysis of 100,000 iterations—a probabilistic computer simulation method—using Equation (1) and (2) in order to estimate the intake of AFM1 from consumption of milk. As numbers of analyzed samples per country were low, triangular concentration distribution was assumed [47]. Random samples from the assumed triangular concentration distribution were used for mycotoxin concentration. Probability distribution fitting for body weight and daily/weekly intake of milk and yoghurt, as well as Monte Carlo simulation, was performed using Minitab. As no distribution identification was achieved when comparing our data with 16 different distributions, visual analysis of the distributions was then considered to assess the fitting of the probability distributions [48].

## Figures and Tables

**Table 1:** Demographic profile of participants sampled (*N* = 1246).

Demographic Characteristics		Total	Serbia	Greece
Gender	Male	523 (42.0%)	281 (37.8%)	242 (48.2%)
	Female	723 (58.0%)	463 (62.2%)	260 (51.8%)
Age	Less than 22 years	648 (52.0%)	270 (36.3%)	378 (75.3%)
	22–27 years	598 (48.0%)	474 (63.7%)	124 (24.7%)
Total		1246 (100%)	744 (100%)	502 (100%)
Average body weight [kg]		68.2 ± 13.8	68.0 ± 13.9	68.4 ± 13.9

**Table 2:** Frequency of milk and yoghurt consumption and average intake of milk and yoghurt in Serbia and Greece (*N* = 1246).

Country	At Least Once A Day (Milk/Yogurt)	At Least Once A Week (Milk/Yogurt)	On A Monthly Basis or Rarer (Milk/Yogurt)	Total
Serbia ^A^	150/203 (20.2%/27.3%)	360/426 (48.4%/57.3%)	234/115 (31.5%/15.5%)	744 (100%)
Greece ^B^	102/28 (20.3%/5.6%)	176/232 (35.1%/46.2%)	224/242 (44.6%/48.2%)	502 (100%)
**Overall**	252/231 (20.2%/18.5%)	536/658 (43.0%/52.8%)	458/357 (36.8%/28.7%)	1244 (100%)
**χ^2^ = 26.524; *p* < 0.05/χ^2^ = 195.319; *p* < 0.05**				
**Gender**				
Male	117/85 (22.4%/16.3%)	238/271 (45.5%/51.8)	168/167 (32.1%/31.9%)	523 (100%)
Female	135/146 (18.7%/20.2%)	298/387 (41.2%/53.5%)	290/190 (40.1%/26.3%)	723 (100%)
**Age**				
Young (<22 years)	137/97 (21.1%/15%)	272/327 (42.0%/50.5%)	239/224 (36.9%/24.6%)	648 (100%)
Older (>22 years)	115/134 (19.2%/22.4%)	264/331 (44.1%/55.4%)	219/133 (36.6%/22.2%)	598 (100%)
**Average daily intake of milk and yoghurt (1-day recall)**	**Per person (mL)**		**Per kg bw (mL)**	
	**Serbia**	**Greece**	**Serbia**	**Greece**
Male	612.9 ± 871.9 ^a^	711.2 ± 1189.2 ^a^	7.6 ± 11.1 ^a^	9.4 ± 16.3 ^a^
Female	503.9 ± 656.0 ^a^	694.4 ± 1156.8 ^a^	8.6 ± 11.7 ^b^	11.5 ± 18.8 ^a^
Total	545.1 ± 746.9 ^A^	702.5 ± 1172.5 ^A^	8.2 ± 11.5 ^A^	10.5 ± 17.7 ^B^
**Average daily intake of milk and yoghurt (7-day recall)**	**Per person (mL)**		**Per kg bw (mL)**	
	**Serbia**	**Greece**	**Serbia**	**Greece**
Male	322.0 ± 211.3 ^a^	463.9 ± 239.1 ^a^	3.9 ± 2.6 ^a^	6.2 ± 3.5 ^a^
Female	300.4 ± 242.4 ^b^	501.4 ± 288.1 ^a^	5.1 ± 4.3 ^b^	8.4 ± 4.9 ^b^
Total	308.6 ± 231.4 ^A^	483.4 ± 266.3 ^B^	4.7 ± 3.8 ^A^	7.4 ± 4.4 ^B^

Note: Items denoted with different small letters are significantly different within the demographic category and items denoted with different capital letters are significantly different between the countries. Statistical significance was set at *p* < 0.05.

**Table 3:** Occurrence of aflatoxin M1 (AFM1) in milk in Serbia and Greece.

Country	Type of Commodity	Number of Samples	Positive Samples	Mean (μg kg^−1^)	Range (μg kg^−1^)	Production Year	Reference
Serbia	UHT/pasteurized milk	42	39	0.270	0.010–0.800	2013	[17]
	UHT/pasteurized milk	104	104	0.200–0.670 *	0.020–1.200	2013	[18]
	Organic milk	6	6	0.030–0.040 *	0.010–0.080	2013	[18]
	UHT/pasteurized milk	20	20	0.133	0.024–0.319	2013	[19]
	UHT milk	223	180	0.071	0.005–>1.000	2013	[11]
	UHT/pasteurized milk	60	54	0.026	0.005–0.104	2014	[19]
	UHT milk	105	17	0.022	0.005–>1.000	2014	[11]
	Heat treated milk	468	364	0.027	<0.005–0.278	2015	[16]
	Heat treated milk	765	753	0.039	<0.005–0.280	2016	[16]
	Total	1793	1537				
Greece	Refrigerated milk	32	NR	NR	0.0002-0.018	2016	[22]
	Pasteurized	82	70	0.010	<0.005–0.05	1999–2000	[21]
	UHT	17	14	0.020	<0.005–0.05	1999–2001	[21]
	Conventional	154	75	0.009	0.006–0.013	2012	[20]
	Total	285	159				

UHT, Ultra-Heat Treatment; NR, Not Reported; * Depending on product type and producer.

**Table 4:** Estimated daily dairy-borne intake of aflatoxin M1 (AFM1) and risk characterization for the two countries.

AFM1 Intake and Risk Characterization	AFM1 ng/kg bw/Day (1-Day Recall) *		AFM1 ng/kg bw/Day (7-Day Recall) *		Range of HCC Cases/Year/10^5^ Individuals (1.2–2.6% of HBsAg+) (1-Day Recall)		Range of HCC Cases/Year/10^5^ Individuals (1.2–2.6% of HBsAg+) (7-Day Recall)		Range of HI Values (1–7-Day Recall)		MOE Values (1–7-Day Recall)	
Country	Serbia	Greece	Serbia	Greece	Serbia	Greece	Serbia	Greece	Serbia	Greece	Serbia	Greece
**Mean**	2.674	0.499	1.238	0.350	0.0036–0.0047	0.0007–0.0009	0.0017–0.0022	0.0005–0.0007	13.4–6.2	2.5–1.8	213.2–460.4	1142.3–1628.6
**5th percentile**	0.302	0.078	0.144	0.080	0.0004–0.0005	0.0001–0.0001	0.0002–0.0003	0.0001–0.0001	1.5–0.7	0.4–0.4	1887.4–3958.3	7307.7–7125
**1st quartile**	0.933	0.203	0.448	0.161	0.0013–0.0016	0.0003–0.0004	0.0006–0.0008	0.0002–0.0003	4.7–2.2	1.0–0.8	610.9–1272.3	2807.9–3540.4
**3rd quartile**	3.636	0.673	1.675	0.461	0.0049–0.0064	0.0009–0.0012	0.0023–0.0029	0.0006–0.0008	18.2–8.4	3.4–2.3	156.8–340.3	846.9–1236.4
**95th percentile**	7.841	1.353	3.698	0.873	0.0106–0.0138	0.0018–0.0024	0.0050–0.0065	0.0012–0.0015	39.2–18.5	6.8–4.4	72.7–154.1	421.3–652.9

* All values were derived from a Monte Carlo simulation.
